# Rosemary Essential Oils as a Promising Source of Bioactive Compounds: Chemical Composition, Thermal Properties, Biological Activity, and Gastronomical Perspectives

**DOI:** 10.3390/foods10112734

**Published:** 2021-11-09

**Authors:** Darko Micić, Saša Đurović, Pavel Riabov, Ana Tomić, Olja Šovljanski, Snežana Filip, Tomislav Tosti, Biljana Dojčinović, Rade Božović, Dušan Jovanović, Stevan Blagojević

**Affiliations:** 1 Institute of General and Physical Chemistry, Studentski trg 12/V, 11158 Belgrade, Serbia; micic83@gmail.com (D.M.); stevan.blagojevic@gmail.com (S.B.); 2 Federal State Budgetary Scientific Institution GOSMETODCENTR, Lusinovskaya 51, 115093 Moscow, Russia; paryabov88@gmail.com; 3 Faculty of Technology, University of Novi Sad, Bulevar Cara Lazara 1, 21000 Novi Sad, Serbia; anav@uns.ac.rs (A.T.); oljasovljanski@uns.ac.rs (O.Š.); 4 Technical Faculty “Mihajlo Pupin” Zrenjanin, University of Novi Sad, Djure Djakovica bb, 23000 Zrenjanin, Serbia; filipsnezana@gmail.com; 5 Faculty of Chemistry, University of Belgrade, Studentski trg 12, 11158 Belgrade, Serbia; tosti@chem.bg.ac.rs; 6 Institute of Chemistry, Technology and Metallurgy—National Institute of the Republic of Serbia, University of Belgrade, Njegoševa 12, 11000 Belgrade, Serbia; bmatic@chem.bg.ac.rs; 7 Faculty for Hotel and Tourism Management in Vrnjačka Banja, University of Kragujevac, Vojvodjanska ulica bb, 36210 Vrnjačka Banja, Serbia; bozovicr@gmail.com; 8 Faculty of Agriculture, University of Novi Sad, Trg Dositeja Obradovica 8, 21000 Novi Sad, Serbia; dusan.jov87@gmail.com

**Keywords:** rosemary essential oil, gastronomical perspectives, chemical composition, biological activity, DSC

## Abstract

Rosemary (*Rosmarinus officinalis* L.) is a plant worldwide cultivated mainly for essential oils, extracts, and as a spice. Up-to-date results showed diversity in composition of the essential oils, which may influence their quality, biological activity, and thermal properties. Therefore, the aim of this study was to investigate the chemical composition, antimicrobial activity, and thermal properties of the rosemary essential oils originating from Serbia and Russia. Additionally, oils were added to the sunflower oils in order to investigate possible antioxidant activity during the frying. Investigation of the chemical profile marked α-pinene, eucalyptol, and camphor as the most abundant compounds in both oils. However, overall composition influenced in such manner that Russian oil showed significantly higher antimicrobial activity, while Serbian oil proved to be better antioxidant agent in case of frying of sunflower oil. This would significantly influence possible application of the oils, which could be used as an antioxidant agent for extension of the food shelf life, or antimicrobial agent for protection against different microbial strains.

## 1. Introduction

*Rosmarinus officinalis* L. (rosemary) is the plant from *Lamiaceae* family, genus *Rsomarinus* L. [1,2]. This plant is cultivated worldwide due to its essential oils, extracts, as a spice, and due to different biological activities [3]. Essential oils of this plant possess many pharmacological properties [2]. When it comes to the chemical profile, there are differences connected to the regionality, seasonality, environmental conditions, agronomic conditions, and varieties in rosemary itself [1,2,3,4]. In most cases, α-pinene, eucalyptol, and camphor are major compounds in rosemary essential oil [1,2,4,5]. However, other compounds such as verbenone, borneol, camphor, and bornyl acetate were also reported as one of the principal compounds in the essential oil [6,7,8]. Consequences of such discrepancies in the chemical composition may be different level of biological activity and differences in behavior, i.e., thermal and other properties. These variations may significantly influence the quality and possibility of application of the essential oil. Therefore, it is very significant to monitor chemical composition during the prolonged period (years) in order to provide high quality of the oils for the market [2].

Sunflower oil is one of the most important and most common edible oils in the world together with soyabean, rapeseed, and cottonseed oils [9,10]. Although it is commonly used for the preparation of the food, this oil is susceptible to oxidation especially due to the presence of unsaturated fatty acids, e.g., linoleic and linolenic acids [11,12]. Oxidation may cause rancid odor, unpleasant flavor, discoloration, and many other products due to the secondary oxidation processes which further decrease nutritional quality and safety of the food [10,11,13]. Occurrence of the oxidation processes in the oils is influenced by several factors such as oxygen exposure, light, temperature, occurrence of the metals, e.g., Fe and Cu [14]. In order to prevent these processes and to prevent shelf-life of the sunflower oil, synthetic and natural antioxidants are added in [11,12]. The most common are butylated hydroxytoluene (BHT), butylated hydroxyanisole (BHA), and tertiary butyl hydroquinone (TBHQ) [12]. However, natural occurring antioxidants attract more and more attention due to the unwanted side effects of synthetic antioxidants [15]. Compounds such as polyphenolics and terpenes are potent antioxidants able to scavenge lipid radicals and to chelate metal ions [12,14]. Due to the well-known antioxidant activity of the essential oils, they were chosen as the natural antioxidant agent and their influence on the shelf-life was investigated by several research groups [9,10,11,12,16].

Because of the above-mentioned importance of monitoring the chemical composition and thermal properties of the essential oils, this study aimed to investigate chemical composition and thermal properties of the commercial essential oils from Serbia and Russia. For such purpose, Serbian and Russian rosemary essential oils were analyzed for chemical profile by the gas chromatography coupled with the mass spectrometry (GC/MS) and inductively coupled plasma coupled to the optical emission spectroscopy (ICP-OES). Thermal properties were investigated by differential scanning calorimetry (DSC). Additionally, biological activity was assessed by using four bacteria strains, one yeast, and one fungi strain. After the initial assessment, essential oils samples were added to the sunflower oil in different amounts for investigation of the possibility of their application as antioxidant agent during the frying process. Frying process was simulated on the DSC at the isothermal conditions at 140 °C. To the best of our knowledge, this is the first time that DSC was used in such a study. Ability of the rosemary EO to scavenge free radicals could be successfully used for extension of the shelf life of the sunflower oil and/or to protect it from generation of radical species during the exposure to the elevated temperatures. Moreover, antimicrobial activity may also be a useful characteristic for possible application of the oil as a natural protective agent. Therefore, results presented herein will be of high importance for further implementation of essential oils.

## 2. Materials and Methods

### 2.1. Chemicals and Reagents

Both rosemary oils are available on the marked and obtained directly from the manufacturer. Serbian oil (SRB) was purchased from Herba Oils (Belgrade, Serbia). Russian oil (RF) was acquired from NPF Carstvo Aroma (Crimea, Russia). Sunflower oil was made by Dijamant DOO (Zrenjanin, Serbia) and is commercially available. All terpenes’ standards were purchased from Sigma Aldrich and were analytical standard grade (≥99%). Methylene chloride was acquired from Centrohem and was of analytical grade purity.

### 2.2. GC/MS Analysis

Analysis of essential oil (EO) samples was done with ion trap GCMS (Thermo Fisher, MA, USA). The analysis was performed using the well-known and described method [17,18]. TR WAX-MS (30 m × 0.25 mm, 0.25 μm) capillary column was used, while analyzed samples were dissolved in methylene chloride and injected into GC through TriPlus AS autosampler (2 μL). Temperature program was as follows: initial temperature 45 °C (8 min), then 8.0 °C/min to 230 °C (10 min). Carrier gas was helium (1 mL/min), while injector was operated in split mode (80:1). Injector, MS transfer line, and ion source temperatures were 250 °C, 200 °C, and 220 °C, respectively. Data acquisition was conducted in *m*/*z* range of 30–300. Compounds were identified combining the NIST 08 MS database and MS spectra of analyzed standards (matching factors were higher than 850). Final results were expressed as relative percentage (%). Quantitative analysis was performed by creating the calibration curves for analyzed compound in concentration range of 1.0–500.0 μg/mL. The final content of compounds was expressed as milligram per gram of EO (mg/g EO).

### 2.3. Contents of Major and Trace Elements

The digestion of the sample of essential oils was performed on microwave system for digestion (Advanced Microwave Digestion System, Ethos 1, Milestone, Italy) equipped with the HPR-1000/10S high pressure segmented rotor. About 0.5 g of sample was precisely weighed with accuracy ± 0.1 mg in placed in quartz inserts and mixed with of 5 mL HNO3 (65 wt.%, Suprapur^®^, Merck KGaA, Darmstadt, Germany). Temperature program of the microwave oven was as follows: increasing in the temperature up to 180 °C for 15 min, maintaining it in the next 20 min, following by the rapid decreasing to the room temperature. Obtained solution was further diluted with the ultrapure water to 25 mL in a volumetric flask. Presence and content of elements and minerals in the samples were established by the ICP-OES (iCAP 6500 Duo ICP, Thermo Fisher Scientific, Cambridge, UK). Concentrations of elements of sample was expressed as mg/kg.

### 2.4. Thermal Analysis

The Q1000 Differential Scanning Calorimeter and Q500 Thermogravimetric Analyzer (TA Instruments, New Castle, DE, USA) were used for thermal analysis of RF and SRB EO. Acquired thermograms were analyzed using TA Advantage Universal analysis 2000 software (version 5.5.24).

#### 2.4.1. Thermal Characterization of EO

The temperature range of the conducted DSC experiments was from 0 to 350 °C. All experiments were conducted under the inert atmosphere (nitrogen) at flow of 50 mL/min. Samples (3.0 ± 0.3 mg) were heated at the rate of 5 °C/min in hermetic Al pans. Thermogravimetric experiments were performed in non-isothermal and isothermal conditions. Samples were weighted in 10.0 ± 0.5 mg. Experiments were also conducted under the inert atmosphere (nitrogen with the flow of 60 mL/min). In the experiments at the non-isothermal conditions the samples were heated to 160 °C at the rate 5 °C/min. In isothermal conditions the samples were kept at 60 °C. Friedman’s non-isothermal isoconversional methods [19] was used calculate the activation energy (Ea) of the evaporation of the tested EOs. Five heating rates were used for this purpose (2, 5, 10, 15, and 20 °C/min). ICTAC Kinetics Committee recommendations for collecting kinetic data [20] and for performing kinetic computations [20] were followed when performing kinetic studies.

#### 2.4.2. Oxidative Stability of Sunflower Oil with Different Share of EO

In order to examine the effect of adding EOs on the oxidative stability of sunflower oil, five concentrations (0.1, 0.5, 1, 5 and 10% (*w*/*w*)) of RF and SRB EO in sunflower oil were made. Sunflower oil without the addition of EO was used as a control sample. The oxidative stability of all sunflower oil samples was determined by measuring the oxidation induction time (OIT) using the DSC method [21], at 140 °C and under oxygen flow of 50 mL/min. Open aluminum pans were used, and mass of samples was 3.0 ± 0.3 mg. OIT represents the time from the heating of the oil sample at a certain isothermal temperature to the beginning of the oxidation process in it. The higher OIT values indicate that the analyzed oil sample is more oxidatively stable.

### 2.5. Antimicrobial Activity of Samples

In this study, the antimicrobial activity of the tested R. officinalis EO was tested against four bacteria—two Gram negative: *E. coli* (ATCC 25922) and *P. aeruginosa* (ATCC 27853) and two Gram positive: *B. cereus* (ATCC 11778) and *S. aureus* (ATCC 25923). Moreover, the antimicrobial potential of the selected oils on eukaryotic type of cells was examined on *S. cerevisiae* (ATCC 9763) and *A. brasiliensis* (ATCC 16404). All strains were obtained from the American Type Culture Collection and the cultures were kept frozen at −80 °C in cryovials with the addition of glycerol as a cryoprotectant.

For the assessment of the antimicrobial activity of the R. officinalis EO two methods have been employed: disc diffusion method and microdilution method for determination of minimum inhibitory concentration (MIC). Both methods were previously described in details [22,23].

Bacterial strains were grown on Müller-Hinton agar (HiMedia, Mumbai, India) at 37 °C for 24 h and at 30 °C (Bacillus cereus ATCC 11,778 and Bacillus cereusw) for 18 h. Yeast strains were grown on Sabouraud Maltose agar (HiMedia, Mumbai, India) at 25 °C (Saccharomyces cerevisiae ATCC 9763) or at 37 °C (Candida albicans ATCC 10231) for 48 h. Cells were suspended in a sterile 0.9% NaCl solution. Suspensions were adjusted to a concentration of 1·106 cfu/mL (estimated by Densichek; BioMérieux, Marcy-l’Étoile, France). Afterwards, 2 mL of the prepared suspensions for inoculation were homogenized with 18 mL of melted (45 °C) media (the same as for suspension preparation) and poured into Petri dishes. After the solidification, four sterile discs (6 mm in diameter) (HiMedia, Mumbai, India) were placed onto the previously inoculated agar plates. Applied disks were impregnated with 15 μL of the EO dissolved in dimethyl sulfoxide (50 mg/mL). Dimethyl sulfoxide was used as negative control, while chloramphenicol, tetracycline, and actiodion were used as a positive control After the incubation period, the diameter of the inhibition halo zone was measured for each disk using HiAntibiotic Zone Scale™ (HiMedia, Mumbai, India). Each experiment was performed in triplicate (*n* = 3).

Minimal inhibitory concentration was assessed for gram-positive bacteria using the microdilution method in sterile flat-bottom 96-well microtiter plates. The preparation procedure of suspensions for inoculation is previously described Disk diffusion method. 1 mL of the prepared suspension (1 × 10^6^ cfu/mL) was homogenized with 9 mL of Müller-Hinton broth (HiMedia, Mumbai, India). In order to obtain final concentration in each well (*n* = 3), 100 μL of inoculated media were mixed with 100 μL of extract dilutions. In each test microtiter plate, there were a positive control (inoculated media without extracts) and a negative control (100 μL of medium mixed with 100 μL of extracts). All test plates were incubated for 24 h at 37 °C or at 30 °C (Bacillus strains). Afterwards, a 100 μL aliquot was poured into Petri dishes and homogenized with Plate Count agar (HiMedia, Mumbai, India). Petri dishes were incubated under identical conditions as microtiter plates and the colonies were enumerated by viable count following the incubation period.

Minimal inhibitory concentration (MIC) is known as the lowest concentration of antimicrobial agent that, under defined in vitro conditions, prevents the appearance of visible growth of a microorganism within a defined period of time. MIC is usually calculated as 100 × (Nc − Nt)/Nc (%), where Nc and Nt are numbers of cells of positive control and treatment, respectively.

### 2.6. Statistical Analysis

All measurements in this study had been performed in triplicates. *t*-test and analysis of variance (ANOVA) followed by Tukey’s HSD test (*p* < 0.05) were used for the statistical analysis. In the OIT results, it was analyzed whether the adding EO had an antioxidative or prooxidative effect compared to sunflower oil without EO, how the concentration of added EO affected the OIT values, and how the type of EO affected the OIT values. All samples that had a statistically significantly higher or lower OIT value (antioxidative and prooxidative effect, respectively) than pure sunflower oil were marked with an asterisk in superscript. Different uppercase letters in the same EO indicate a significant difference of the OIT depending on the concentration of the added EO. Different lowercase letters in the same concentration of the added EO indicate a significant difference of the OIT depending on the type of EO. XLSTAT (version 2014.5.03, Addinsoft, New York, NY, USA) and statistics add-in for MS Excel were used to perform above-mentioned statistical calculations.

## 3. Results

### 3.1. Chemical Profiles of Essential Oils

Both Serbian (SRB) and Russian (RF) rosemary oils were analyzed for assessment of chemical profile and composition of terpenes, minerals, and elements. Result of the GC/MS analysis is given in Table 1, while chromatograms are given in Figure S1 (Supplementary Data).

Obtained results showed that three compounds were principle in both essential oils: α-pinene (23.00% and 17.76% in SRB and RF oils, respectively), eucalyptol (17.79% and 23.40 in SRB and RF oils, respectively), and camphor (14.39% and 17.17% in SRB and RF oils, respectively). Both eucalyptol and camphor were found in higher percentage in RF compared to the SRB oil. However, quantification showed that all three compounds were presented in higher amount in SRB oil. Beside above-mentioned compounds, there were several compounds detected in higher amount: camphene, β-pinene, limonene, p-cymene, borneol, bornyl acetate, and trans-β-caryophyllene.

Results also showed certain diversity in chemical profile between the samples. Thus, α-terpineol and terpinen-4-ol were found only in SRB sample. Moreover, several other compounds, such as γ-terpinene, α- and β-phellandrenes, terpinolene, were also found only in SRB oil sample. On the other hand, m-cymene, α-pinane oxide, pinocarvone, carvone, and several other compounds were detected in RF sample (Table 1). It is expected that this diversity in profile would influence their behavior and biological activity.

Previously reported studies about the chemical composition of rosemary essential oils showed also certain diversity in chemical profile and composition. Pellegrini et al. (2018) found camphor to be the principal compound (22.07%) followed by α-pinene (16.64%), eucalyptol (15.71%), and borneol (11.99%) [5]. Interestingly, authors reported absence of limonene in this sample, while borneol was significantly higher than in our samples. Investigation of seasonal diversity in composition of the rosemary oil showed changes in the content and overall profile of analyzed samples [2]. Despite these changes, camphor was reported as the principal compound (35.93–24.38%) followed by eucalyptol (19.26–22.68%), and myrcene (9.55–15.25%). Similar results were obtained by Zaouali et al. [1], but with eucalyptol as the principal compound in most cases. Bajalan et al. (2007) investigated composition of rosemary oils isolated from seven Iranian populations of this plant. However, despite differences in EO’s sources, authors confirmed the prevalence of camphor, eucalyptol, and α-pinene [4]. The same case was for study reported by Jordan et al. (2013), where authors investigated influence of phenological stage on chemical composition of rosemary essential oil. Major compounds were the same, i.e., α-pinene (13.0–15.5%), eucalyptol (18.9–21.2%), and camphor (17.0–18.6%) [3]. Bousbia et al. (2009) applied two different approaches for isolation of essential oil, i.e., hydrodistillation and microwave hydrodifussion and gravity, and compared chemical profile of obtained samples. Results were similar when comparing to each other, where α-pinene was the principal compound, followed by camphor and verbenone. However, authors did not report presence of eucalyptol, which is one of the main compounds in our samples [8]. Karakaya et al. (2014) also investigated effects of different extraction techniques (hydrodistillation and microwave-assisted hydrodistillation) on the composition of the rosemary oil. They found eucalyptol to be the principal compound, followed by camphor, α-pinene, borneol, and camphene [7]. There is also study of composition of commercial essential oil [6], which also reported camphor to be the main compound (35.5%), followed by eucalyptol (18.2%). Surprisingly, authors reported rather high content of bornyl acetate (13.4%) and lower content of α-pinene (4.9%) comparing to the results from this study (Table 1).

Although most studies reported the same major compounds, there were other studies which reported slightly different composition. Thus, Bozin et al. (2007) reported limonene (21.7%) and camphor (21.6%) as the principal compounds in essential oils sample [24]. Authors detected eucalyptol in 2.1%, but found linalool oxide in 10.8%. Camphene was reported in lower percentage (3.9%), as well as β-pinene (1.1%). Besides content, samples were differing in composition, where authors reported compounds which were not found herein [24]. Gachkar et al. (2007) also reported significantly different composition of rosemary oil [25]. Piperitone was the main compound (23.7%) followed by α-pinene (14.9%) and linalool (14.9%). Content of eucalyptol and camphor were 7.43% and 4.97%, respectively, which is significantly lower content comparing to our findings (Table 1).

Elements and mineral’s content are given in Table 2. It might be seen that SRB was quite rich in Fe, Ca, Na, and S, while RF was rich in Ca, Na, and S. Comparing the results of these elements, SRB was richer in their content. Arsenic was not found in both samples, while Co was found only in SRB sample in trace level (0.032 mg/kg). Furthermore, Cd and Pb were also detected in trace levels, what makes these oils safe to use in a diet or as a supplement.

There are several available classifications of elements. One of them classifies elements into four major groups: essential, beneficial, contaminating, and polluting elements [26]. According to Stephanos and Addison essential elements are certain nonmetals (C, H, O, N, S, P, Cl, and I), alkali and alkaline-earth metals (Na, K, Mg, and Ca), and transition elements such as Fe, Zn, Mn, Cu, Co, and Mo. In the group of beneficials are different nonmetals, metalloids, and metals (F, Br, Se, Si, Sn, V, Cr, and Ni. Polluting elements are Hg, Cd, and Pb. Presence of certain elements, such as Fe, Ca, Cr, and Mg, is essential for nutritive value of the essential oils. Bulk elements are necessary for the proper functioning of the organism and should be intake at the daily levels. Iron is an essential microelement necessary for hemoglobin and myoglobin synthesis. Besides those two proteins, iron is also essential for cytochromes and some other enzymes. The deficiency of this element is known as anemia. Several types of enzymes require zinc for proper functioning. These are hydrolases, peptidases, and oxidases. This element has also significant role in gene expression and fold stabilization which requires zinc fingers [27]. Copper has also important role in metabolism, i.e., in the electron transfer process in Type III heme-copper oxidases and also Type I blue-copper proteins [28]. All these elements should be ingested daily. Because of such importance, daily intake levels are defined and known as dietary reference intake (DRI) created by the US Department of Agriculture [29]. According to the DRI, daily intake of Na and K, and Ca is measured in grams, while intake of Mg should be in milligrams. Daily intake of phosphorus is 1.25 g/day for both male and female up to 18 years old. After this age, uptake of this element should be lower (700 mg/day). Iron, zinc, and manganese should be also taken in milligrams a day levels, while copper and chromium should be ingested in micrograms a day levels [29].

### 3.2. Antimicrobial Activity of Essential Oils

The next step of this study was to investigate whether the variation in chemical composition and geographical origin of the tested R. officinalis EO affect their antimicrobial potential. Preliminary screening of the in vitro antimicrobial activity was performed by disc-diffusion method. According to the obtained results (Table 3) it might be noticed that RF showed far better antimicrobial potential in comparison to the SRB. In the case of RF, the maximum inhibition zone of 40.00 mm was registered for all tested microorganisms, except for *P. aeruginosa* (21.33 mm) and *A. brasiliensis* (33.00 mm) where the activity might be estimated as moderate to high. Additionally, it should be pointed out that the inhibition zone of RF was even higher than those of the used positive controls (chloramphenicol 30 μg/disc, tetracycline 30 μg/disc, and actidion 30 μg/disc), indicating the possibility of using RF as a natural ingredient in combating the microbial and antimycotic resistance toward the antibiotics. A high antimicrobial activity (above 30.00 mm) was also observed for SRBR against *E. coli*, *S. aureus,* and *S. cerevisiae*. However, this oil showed low to moderate activity against the other tested microorganisms. According to the relevant researches conducted in this area, it can be concluded that rosemary EO usually demonstrated moderate activity against tested set of microorganisms [1,2,6], which is consistent to the results obtained for SRB. To the best of our knowledge, such a high antimicrobial performance of rosemary EO as in the case of RF has not been previously published elsewhere. In the available literature, there are opposite opinions about the carriers of antimicrobial activity in EOs. Some of the authors claim that the dominant chemical components are essential for antimicrobial properties such as camphor, eucalyptol, and α-pinene [1,30]. On the other hand, there are studies which emphasize the importance of minor components in EOs as well as the synergistic effect between terpenoid and phenolic compounds which could be able to disrupt cellular membrane and inhibit cell respiration and ion transport process [3,31].

When it comes to the composition-activity relationship, i.e., structure-activity dependence, it was shown that isomerism does not influences the antimicrobial activity. It has been also reported that functional group’s position does not affect the activity. However, occurrence of the hydroxyl functional group in the structure has significant impact on the antimicrobial activity [32]. Therefore, alcohols are more active than aldehydes [33,34]. Furthermore, terpinen-4-ol proved to be a more potent antimicrobial agent than α-terpineol. Explanation for such behavior is the capacity for creating the hydrogen bonds. In this case, position of OH group in 4-terpineol increases the capacity for making the hydrogen bond [32]. Cyclic monoterpenes β-pinene and limonene also showed significant activity. Thus, β-pinene influences the respiration and leakage of the potassium and hydrogen ions in yeasts [35], while both compounds inhibit energy-dependent processes, such as respiration, in S. cerevisiae [36]. Certain terpenes, e.g., α- and β-pinene, γ-terpinene, and limonene, induce structural and functional changes of the membrane [37]. Previous results indicated that certain properties of the compounds, such as hydrophobicity and lipophobicity, also significantly influence on their antimicrobial potency. These properties allow them to penetrate through the membrane consequently changing the fluidity, permeability, protein properties, etc. [38]. There are reports which indicate that mixture of two or several terpenes showed higher activity than each one separately [39]. Therefore, synergistic effect should be also taken into an account when comparing the activity of these oils, especially because their chemical composition is different, i.e., different compounds were found in SRB and RF. Thus, it has been reported that p-cymene increases the antimicrobial activity of other compounds [34]. This could be, besides synergy, one of the possible explanations for significantly higher activity of RF comparing to the SRB oil.

Besides the chemical composition, some papers confirm the influence of geographical origin [3,4], seasonal variations [2], as well as varieties in rosemary [1,4] on the antimicrobial performance of rosemary’s EO.

After the satisfactory results of preliminary examination by disk-diffusion method, microdilution methods were applied for further investigation of the antimicrobial activity. From the results presented in Table 4 it could be noted that SRB showed good antimicrobial activity against bacteria (MIC ≤ 50%), while in the case of the eukaryotic microorganisms the activity was moderate (MIC > 50%). Moderate activity against eucaryotic organisms could be attributed to their complex cell structure [40]. In contrast, a very low MIC (below 6.3%) of RF was noted for all selected microorganisms. The obtained results indicating a high antimicrobial activity of SRB and RF are in a good correlation with previously reported studies [2,3]. Such a great antimicrobial performance of the tested EO may contribute to their use in reducing foodborne pathogens and extending shelf life of food products or as a potential natural and green replacement of synthetic antibiotics, antimycotics, and preservatives in food and cosmetics industry.

### 3.3. Thermal Analysis of Essential Oils

#### 3.3.1. Thermal Properties

**Figure 1:**
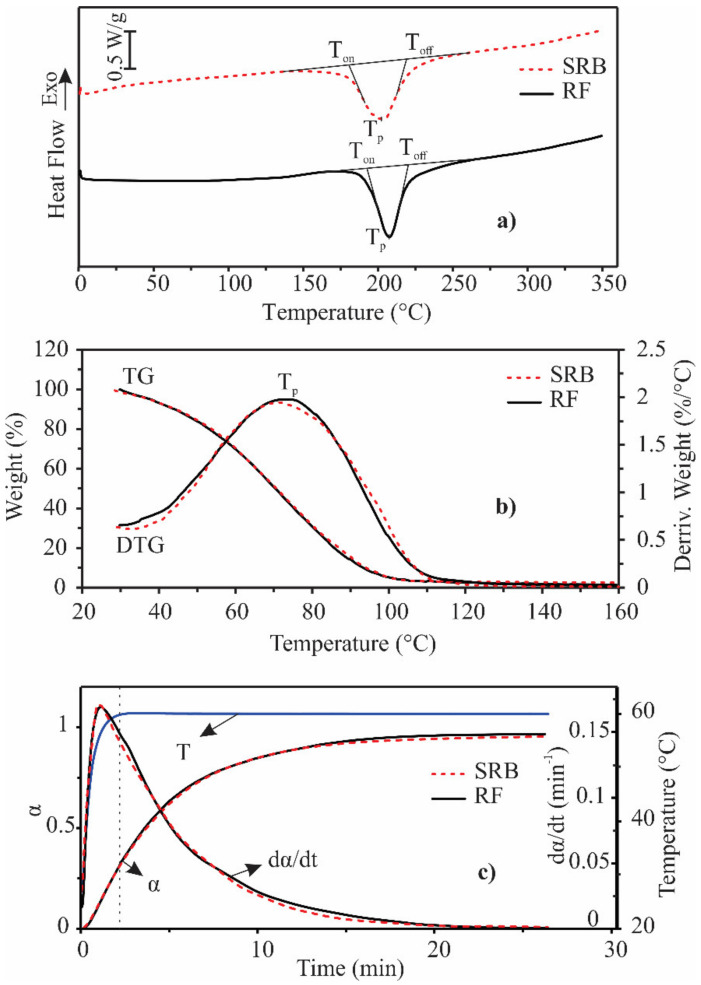
(**a**) DSC and (**b**) TG/DTG curves of Russian (RF) and Serbian (SRB) rosemary essential oils in non-isothermal condition at heating rate of 5 °C/min; (**c**) degree of conversion (α), evaporation rate (dα/dt) and temperature (T) as a function of time in isothermal condition at 60 °C for RF and SRB rosemary essential oils (beginning of isothermal conditions is marked by a vertical dashed line). Abbreviations: DSC—differential scanning calorimetry, TG—thermogravimetry, DTG—differential thermogravimetry.

After the initial evaluation (chemical composition and antimicrobial activity), the next step was to determine the thermal properties. These are quite important data because application of these oils would depend on their stability and evaporation. Obtained results are listed in Table 5, and corresponding curves are shown in Figure 1a. The shape of all curves was almost identical for both EOs indicating that analyzed EOs have similar thermal characteristics. This is rather expected given that the most prevalent components were the same in both EOs (Table 1). One wide endothermic peak, in the range of about 150 to 250 °C for SRB and about 170 to 260 °C for RF, appeared on both DSC curves, which corresponded to the process of evaporation [17,22]. The main step of this process (temperature range from Ton to Toff) of the SRB EO was wider than one of RF EO, which was expected, because more components were detected in SRB by GC-MS analysis. The evaporation process in SRB EO began at a lower temperature than in RF EO (Ton,SRB < Ton,RF, *p* < 0.05), and in both EOs it ended at approximately the same temperature (*p* < 0.05). The boiling temperatures of the predominant components of both samples varied from about 166 to 264 °C [41], which is in accordance with a temperature range of the EOs evaporation process determined by the DSC method.

One mass loss and one peak were detected on the TG and differential TG curves for both samples, respectively, indicating that the evaporation occurred in one step (Figure 1b). Both EOs evaporated completely to about 120 °C, with the residue of 1.5 to 2%. The peak temperature (T_p_) on the DTG curve represents the temperature at which the evaporation process was fastest. T_p_ of SRB EO was lower than T_p_ of RF EO (Table 1, *p* < 0.05), indicating that the evaporation process in SRB EO reached a maximum rate at a lower temperature compared to RF EO. This is consistent with the DSC results that the evaporation process in SRB EO begins at a lower temperature compared to RF EO.

Thermal characteristics of analyzed EOs were also examined in isothermal conditions at 60 °C (Figure 1c). The EOs showed almost identical thermal properties under these conditions, too. About 35% of both EOs evaporated by the time the isothermal conditions were reached (about 2.5 min). Both EOs completely evaporated in about 25 min (extent of conversion—α reached value 1). The rate of the evaporation process under isothermal conditions (dα/dt) was maximal at the beginning and decreased with time, indicating a decelerating type of kinetic model for the process of the evaporation [42]. The explanation for such behavior is that in the beginning, more volatile components evaporate. As they leave the system, less volatile compounds persist in the EOs over time, causing the evaporation rate to decrease. This is in accordance with the literature data for laurel, sage, and coriander EOs, whose evaporation process demonstrated a decelerating type of kinetic model, too [17,22].

**Figure 2:**
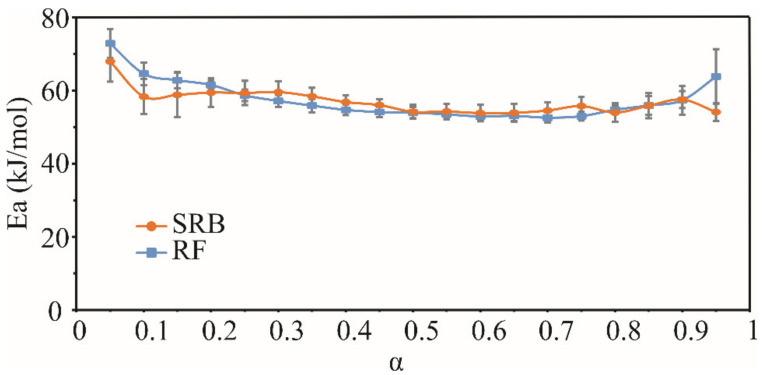
Activation energies (Ea) of the evaporation process of Russian (RF) and Serbian (SRB) rosemary essential oils as a function of conversion degree (α).

The activation energies (Ea) of the evaporation obtained by the Friedman method [19] were in range from 52.5 to 72.6 kJ/mol for RF EO, and from 53.8 to 67.9 kJ/mol for SRB EO. In the literature, the activation energy of evaporation for pure substances is associated with the enthalpy [43,44]. The enthalpies of evaporation of the most prevalent compounds ranged from 37.9 to 52.8 kJ/mol for both oils [41]. These values are slightly lower than the experimentally obtained activation energies of the essential oils’ evaporation process. However, it should be kept in mind that essential oils are complex systems consisting of dozens of different components. These components in the system can physically interact, which can affect their evaporation process. Therefore, they can affect the activation energies of the essential oil evaporation process, which should essentially be the average value of the evaporation enthalpies of individual components of essential oil. Ea values did not vary significantly with the increase in the extent of conversion (α), for both EOs (Figure 2).

Such results imply that the process of the evaporation is a single-step process, which is consistent with the non-isothermal TGA results. The average activation energies in the tested EOs, 57.5 ± 5.4 kJ/mol for RF and 56.9 ± 3.4 kJ/mol for SRB, were not significantly different from each other (*p* < 0.05), which is another confirmation that these oils have very similar thermal properties.

#### 3.3.2. The Effect of Added RF and SRB EO on the Oxidative Stability of Sunflower Oil

**Figure 3:**
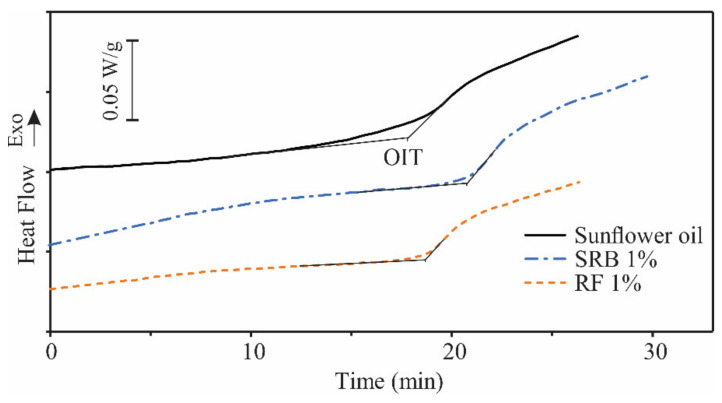
Isothermal DSC oxidation curves of pure sunflower oil and sunflower oil with added Russian (RF) and Serbian (SRB) rosemary essential oil with share of 1% (*w*/*w*) at 140 °C in oxygen flow 50 mL/min, OIT—oxidation induction time.

Finally, investigated essential oils were added to the sunflower oil in different percentages in order to investigate the possibility of EOs utilization as an antioxidant agent during the frying process. The effect of analyzed essential oils in sunflower oil on its oxidative stability was investigated by determining the oxidation induction time (OIT) using DSC in isothermal conditions at 140 °C. DSC is a suitable technique for this purpose, because it simulates the real conditions of using edible oils at high temperatures during heat treatment of foods. The DSC curves of the oxidation process of pure sunflower oil and oil samples containing 1% EO are shown in the Figure 3.

The effect of five different concentrations (0.1, 0.5, 1, 5 and 10% (*w*/*w*)) was examined, and the obtained results are shown in Table 6. It might be seen that, at concentrations of 0.1, 0.5 and 1% of RF, this oil did not have a significant effect on the OIT values of sunflower oil (*p* < 0.05).

Concentrations of 5 and 10% significantly reduced the OIT value compared to pure sunflower oil (*p* < 0.05). The OIT value was reduced about 1.7 times by adding 5% of RF EO and even 4 times by adding 10% of RF EO, indicating that increasing the concentration of RF EO significantly reduced the quality of sunflower oil in terms of its oxidative stability. In the case of SRB EO, concentrations of 0.1, 5 and 10% did not have a significant effect on OIT values compared to pure sunflower oil (*p* < 0.05). Concentrations of 0.5 and 1% slightly increased the OIT value (*p* < 0.05), indicating that they improved the quality of sunflower oil in terms of its oxidative stability. Based on these results, it can be concluded that the addition of SRB EO in a certain concentration can improve the oxidative stability of sunflower oil, and thus its quality. While the addition of RF EO in smaller concentrations does not affect the oxidative stability of sunflower oil, higher concentrations can significantly impair it. The reason for such a different effect on the oxidative stability of sunflower oil could be the presence of different minor components in analyzed EO, since both EOs, RF and SRB, have a similar content of the predominant components.

Positive effect of rosemary essential oil on stability of hazelnuts and poppy oils was previously reported [45]. There is also report of high protection activity of rosemary oil in sunflower oil against oxidation [46]. However, authors investigated antioxidant influence by periodic determination of peroxide value, which is simple volumetric method (titration) after exposing the samples at 50 °C. In this case, investigation has been performed at 140 °C, which is more suitable and simulates the cooking processes which include sunflower oil. In this case, antioxidant activity is very important for this role. It has been reported that carvone, myrcene, and γ-terpinene scavenge DPPH radicals very quickly. It was also shown that terpenes with conjugated double bonds had very high antioxidant potency [47]. In this case presence of such compounds was noticed in both oils, but SRB showed better activity in this case because of the higher content of these compounds in it.

## 4. Conclusions

Investigation of the influence geographical origin on the chemical profile of the rosemary oil indicated the discrepancies in both composition and in content of the identified compounds in analyzed samples. Although the same compounds were the most abundant one in both oils (α-pinene, eucalyptol, and camphor), both oils had specifical compounds which could be detected in only one sample but not in the other. Such diversity significantly influenced the properties of the oils. Therefore, Russian oil (RF) showed significantly higher antimicrobial activity against all tested strains. On the other hand, when oils were injected into a sunflower oil, Serbian oil (SRB) proved to be more potent as antioxidant agent, while RF did not affect the stability in minor concentrations, but decreased oxidative stability of sunflower oil in higher concentrations. Therefore, application of the essential oil would highly depend on the chemical composition and oils have to be properly investigated prior to decision of the field of application. However, the result presented herein showed high potential of the rosemary as an antioxidant and antimicrobial agent. This implies possible application for different purposes, such as a natural preservative agent, to be used instead of artificial agents which may be harmful for human health.

## Figures and Tables

**Table 1:** Chemical profile, relative content (%), and quantitative profile of Serbian (SRB) and Russian (RF) rosemary essential oil.

Compound	SRB		RF	
	Content (%)	Content (mg/g EO)	Content (%)	Content (mg/g EO)
Cyclic monoterpenes				
Myrcene	2.43	3.9 ± 0.1 ^a^	0.05	0.15 ± 0.02 ^b^
Cyclic monoterpenes				
α-Thujene	0.39	-	0.55	-
α-Pinene	23.00	282 ± 5 ^a^	17.76	122 ± 1 ^b^
α-Fenchene	0.09	-	0.22	-
Camphene	9.99	-	8.36	-
β-Pinene	3.49	49.9 ± 0.9 ^a^	5.19	37.7 ± 0.2 ^b^
α-Phellandrene	0.59	-	N.D. *	-
β-Phellandrene	0.13	-	N.D.	-
α-Terpinene	0.25	26.4 ± 0.3	ND.	-
Limonene	4.18	63.4 ± 0.5 ^a^	3.11	27.4 ± 0.3 ^b^
γ-Terpinene	0.43	5.1 ± 0.1	N.D.	-
Terpinolene	0.48	-	N.D.	-
Acyclic oxygenated mnoterpenes				
Linalool	0.85	15.6 ± 0.2 ^a^	0.84	10.0 ± 0.1 ^b^
Cyclic oxygenated monoterpenes				
Eucalyptol	17.79	177 ± 3 ^a^	23.40	169 ± 2 ^b^
α-Pinene oxide	N.D.	-	0.47	-
Fenchone	0.01	0.02 ± 0.00 ^b^	0.02	0.86 ± 0.05 ^a^
α-Campholenal	N.D.	-	0.58	-
Isothujol	0.02	-	N.D.	-
Camphor	14.39	149 ± 3 ^a^	17.17	104 ± 2 ^b^
Bornyl acetate	2.39	22.5 ± 0.3 ^a^	3.31	19.3 ± 0.1 ^b^
Pinocarvone	N.D.	-	0.20	-
Terpinen-4-ol	1.19	14.8 ± 0.1	N.D.	-
Myrtenal	0.04	-	0.33	-
*cis*-Sabinol	0.08	-	0.14	-
Isoborneol	N.D.	-	0.11	0.61 ± 0.02
α-Terpineol	2.30	51.0 ± 0.5	N.D.	-
Borneol	2.39	20.2 ± 0.2 ^b^	4.56	27.7 ± 0.3 ^a^
Carvone	N.D.	-	0.07	3.86 ± 0.06
Myrtenol	0.21	-	0.25	-
*trans*-Carveol	0.17	-	0.05	-
*p*-Cymene-8-ol	N.D.	-	0.08	-
Verbenone	2.33	-	0.30	-
*cis*-Verbenol	0.15	-	0.25	-
Cyclic aromatic monoterpenes				
*p*-Cymene	4.51	33.6 ± 0.4 ^b^	10.91	41.3 ± 0.4 ^a^
*m*-Cymene	N.D.	-	0.02	0.07 ± 0.01
Sesquiterpenes				
α-Copaene	0.18	-	0.03	-
α-Cubebene	0.12	-	0.07	-
*trans*-β-Caryophyllene	3.22	29.7 ± 0.2 ^a^	0.63	4.06 ± 0.09 ^b^
Humulene	1.22	-	N.D.	-
Caryophyllene oxide	0.47	-	0.97	-
Other				
2-Methyl-3-octanone	0.44	-	N.D.	-
3-Octanol	0.03	-	N.D.	-
1-Octen-3-ol	0.08	-	N.D.	-

The values are presented as mean ± SD, different superscripts within the same row indicate significant differences of means, according to *t*-test (*p* < 0.05). * N.D.—Not detected. “-” not determined.

**Table 2:** Elements and minerals in Serbian (SRB) and Russian (RF) essential oil.

Element (mg/kg)	SRB	RF
Bulk elements		
K	1.14 ± 0.08 ^a^	1.26 ± 0.05 ^a^
Na	8.46 ± 0.15 ^a^	8.17 ± 0.36 ^a^
Mg	1.18 ± 0.09 ^a^	0.89 ± 0.01 ^b^
Ca	7.21 ± 0.23 ^b^	9.90 ± 0.20 ^a^
Trace elements		
Co	0.032 ± 0.001	N.D. *
Cr	9.50 ± 0.02 ^a^	0.025 ± 0.002 ^b^
Cu	0.132 ± 0.009 ^a^	0.041 ± 0.005 ^b^
Fe	5.66 ± 0.04 ^a^	1.58 ± 0.17 ^b^
Li	0.022 ± 0.002 ^a^	0.012 ± 0.001 ^b^
Mn	0.261 ± 0.009 ^a^	0.022 ± 0.002 ^b^
Al	0.83 ± 0.03 ^a^	0.81 ± 0.09 ^a^
Sr	0.020 ± 0.004 ^a^	0.012 ± 0.002 ^a^
Ba	0.05 ± 0.02 ^a^	0.004 ± 0.001 ^a^
Ni	2.06 ± 0.01 ^a^	0.23 ± 0.02 ^b^
Zn	0.048 ± 0.001 ^b^	0.107 ± 0.012 ^a^
Se	0.44 ± 0.07 ^a^	0.47 ± 0.10 ^a^
P	1067 ± 2 ^a^	1053 ± 10 ^a^
S	53.61 ± 0.08 ^a^	6.06 ± 0.04 ^b^
Polluting elements		
Pb	0.064 ± 0.003 ^b^	0.115 ± 0.005 ^a^
As	N.D. *	N.D.
Cd	0.024 ± 0.003 ^a^	0.011 ± 0.001 ^b^

The values are presented as mean ± SD, different superscripts within the same row indicate significant differences of means, according to *t*-test (*p* < 0.05). * N.D.—Not detected.

**Table 3:** Antimicrobial activity of *R. officinalis* EO from Serbia (SRB) and Russia (RF) (mean value diameter of the inhibition zone (mm) including disc (6 mm)) ± standard deviation).

Group	Tested Strains	Disk Diffusion Method (15 µL of EO Concentration 100%)		Positive Control			Negative Control
		Tested Samples of EO		Antibiotic/Antimicotic			DMSO 5%
		SRB	RF	CHL	TET	Actidion	
G(-) bacteria	*E.coli* ATCC 25922	32 ± 3 ^b^	40.0 ± 0.0 ^a^	29 ± 2 ^b^	21.0 ± 0.0 ^c^	-	nd *
	*P. aeruginosa* ATCC 27853	10.0 ± 0.0 ^c^	21 ± 3 ^a^	12.3 ± 0.6 ^bc^	14.7 ± 0.6 ^b^	-	nd
G(+) bacteria	*B. cereus* ATCC 11778	12 ± 1 ^d^	40.0 ± 0.0 ^a^	30.3 ± 0.6 ^b^	28 ± 1 ^c^	-	nd
	*S. aureus* ATCC 25923	32 ± 2 ^b^	40.0 ± 0.0 ^a^	29.7 ± 0.6 ^b^	26.0 ± 0.0 ^c^	-	nd
Yeast	*S. cerevisiae* ATCC 9763	33 ± 2 ^b^	40.0 ± 0.0 ^a^	-	-	40.0 ± 0.0 ^a^	nd
Fungi	*A. brasiliensis* ATCC 16404	11 ± 2 ^c^	33.0 ± 0.0 ^a^	-	-	26.3 ± 0.6 ^b^	nd

Values are presented as mean ± standard deviation (*n* = 3), different lowercase superscript within the same row indicate a significant difference of means according to Tukey’s honest significant difference (HSD) test (*p* < 0.05). CHL-chloramphenicol, TET-tetracycline, DMSO-dimethyl sulfoxide. * nd—not detected.

**Table 4:** Minimal inhibitory concentration (MIC) of rosemary EO from Serbia (SRB) and Russia (RF) assayed by the microdilution method.

Group	Tested Strains	MIC [%]	
		Tested Samples of EO	
		SRB	RF
G(−) bacteria	*E. coli* ATCC 25922	12.5	0.8
	*P. aeruginosa* ATCC 27853	50.0	6.3
G(+) bacteria	*B. cereus* ATCC 11778	12.5	0.8
	*S.aureus* ATCC 25923	6.3	0.8
Yeast	*S. cerevisiae* ATCC 9763	>50.0	1.6
Fungi	*A. brasiliensis* ATCC 16404	>50.0	1.6

**Table 5:** Results of thermal analysis (DSC and TGA) of Russian and Serbian rosemary essential oils.

Parameters	SRB	RF
DSC		
T_on_ (°C)	179 ± 1 ^b^	192 ± 2 ^a^
T_p_ (°C)	204 ± 2 ^b^	207 ± 1 ^a^
T_off_ (°C)	223 ± 2 ^a^	221 ± 1 ^a^
ΔH (J/g)	320 ± 10 ^a^	256 ± 9 ^b^
TGA		
T_p_ (°C)	71 ± 2 ^b^	74 ± 1 ^a^
T_s_ (°C)	29 ± 2 ^a^	30 ± 3 ^a^
T_e_ (°C)	121 ± 2 ^a^	122 ± 2 ^a^
Residue at T_e_ (%)	1.9 ± 0.5 ^a^	1.5 ± 0.3 ^a^

Values are presented as mean ± standard deviation (*n* = 3), different lowercase superscript within the same row indicate a significant difference of means according to Tukey’s honest significant difference (HSD) test (*p* < 0.05). T_on_—onset temperature, T_off_—offset temperature, T_p_—peak temperature, T_s_—start temperature, T_e_—end temperature.

**Table 6:** Oxidation induction time (OIT) of sunflower oil with different share of added Russian (RF) and Serbian (SRB) essential oils.

Percentage of Added EO (% *w*/*w*)	OIT (min)	
	SRB	RF
0	18 ± 1	
0.1	19.4 ± 0.8 ^AB,a^	20 ± 1 ^A,a^
0.5	20.7 ± 0.8 ^AB,a,^*	20.2 ± 0.2 ^A,a^
1	21 ± 1 ^A,a,^*	18.5 ± 0.4 ^A,b^
5	18.3 ± 0.6 ^BC,a^	10.8 ± 0.8 ^B,b,^*
10	16.3 ± 0.8 ^C,a^	5 ± 1 ^C,b,^*

Values are presented as mean ± standard deviation (*n* = 3), different uppercase superscript within the same column indicate a significant difference of means, different lowercase superscript within the same row indicate a significant difference of means, and asterisk (*) indicate a significant difference of means between all samples and sunflower oil without essential oil according to Tukey’s honest significant difference (HSD) test (*p* < 0.05).

## Supplementary Materials

The following are available online at https://www.mdpi.com/article/10.3390/foods10112734/s1, Figure S1: Chromatograms of Serbian (upper) and Russian (bottom) essential oils. TIC values for Serbian and Russian oils were 8.90 × 107 and 6.69 × 107, respectively, Table S1: Retention time, ion fragments, and identification methods for rosemary essential oils.
