# Supplementary material for: Rosemary Essential Oils as a Promising Source of Bioactive Compounds: Chemical Composition, Thermal Properties, Biological Activity, and Gastronomical Perspectives
Source: Foods. 2021 Nov 9;10(11):2734. doi: 10.3390/foods10112734 (PMC8623706; doi:10.3390/foods10112734)
Supplement: Supplementary file 1 [file foods-10-02734-s001.zip › foods-1435543-supplementary.pdf]

Table S1. Retention time, ion fragments, and identification methods for rosemary essential oils.

Compound	RT	Identification framgents (m/z)	Identification methods
α-Thujene	3.79	93, 136	NIST
α-Pinene	4.14	79, 91, 93, 105, 121, 136	STD, NIST
α-Fenchene	4.88	93, 121, 136	NIST
Camphene	5.12	93, 121, 136	NIST
β-Pinene	6.43	79, 91, 93, 105, 121, 136	STD, NIST
α-Phellandrene	8.28	93, 136	NIST
β-Phellandrene	9.02	93, 136	NIST
Myrcene	9.26	39, 41, 91, 93, 107, 121, 136	STD, NIST
α-Terpinene	9.59	93, 121, 136	STD, NIST
Limonene	10.36	67, 79, 93, 107, 121, 136	STD, NIST
Eucalyptol	10.66	81, 93, 108, 139, 154	STD, NIST
γ-Terpinene	11.79	77, 79, 91, 93, 105, 121, 136	STD, NIST
2-Methyl-3-octanone	12.19	43, 71, 99, 142	NIST
p-Cymene	12.52	91, 119, 134	STD, NIST
Terpinolene	12.81	93, 121, 136	NIST
m-Cymene	13.32	91, 119, 134	STD, NIST
α-Pinene oxide	14.88	67, 109, 137	NIST
Fenchone	15.37	41, 79, 81, 152	STD, NIST
3-Octanol	15.49	59, 83, 101, 112	NIST
α-Campholenal	16.32	93, 108, 152	NIST
1-Octen-3-ol	16.58	57, 128	NIST
α-Copaene	16.64	105, 119, 161, 204	NIST
Isothujol	16.81	43, 55, 93, 121, 136, 154	NIST
α-Cubebene	17.27	105, 119, 161, 204	NIST
Camphor	17.68	67, 81, 95, 108, 152	STD, NIST
Linalool	18.26	43, 81, 93, 121, 136, 154	STD, NIST
Pinocarvone	18.54	53, 81, 108, 135, 150	NIST
Bornyl acetate	18.77	95, 108, 121, 137, 154, 196	STD, NIST
trans-β-Caryophyllene	19.03	91, 105, 119, 133, 147, 161, 175, 189, 204	STD, NIST
Terpinen-4-ol	19.12	71, 86, 93, 111, 136, 154	STD, NIST
Myrtenal	19.52	79, 107, 135, 150	NIST
cis-Sabinol	19.92	81, 92, 109, 119, 134, 152	NIST
Isoborneol	20.10	95, 154	STD, NIST
Humulene	20.18	93, 121, 204	NIST
cis-Verbenol	20.28	94, 109, 119, 137, 152	NIST
α-Terpineol	20.60	81, 93, 121, 136, 154	STD, NIST
Borneol	20.70	67, 95, 121, 137, 154	STD, NIST
Verbenone	20.74	91, 107, 135, 150	NIST
Carvone	21.17	39, 54, 82, 93, 108, 122, 135, 150	STD, NIST
Myrtenol	21.92	79, 91, 108, 119, 152	NIST
trans-Carveol	22.53	84, 109, 119, 152	NIST
p-Cymene-8-ol	22.73	43, 135, 150	NIST
Caryophyllene oxide	24.52	43, 79, 93, 109, 121, 220	NIST

NIST-identification by comparation with spectrum in NIST database, STD-identification by comparation with spectrum of the standard

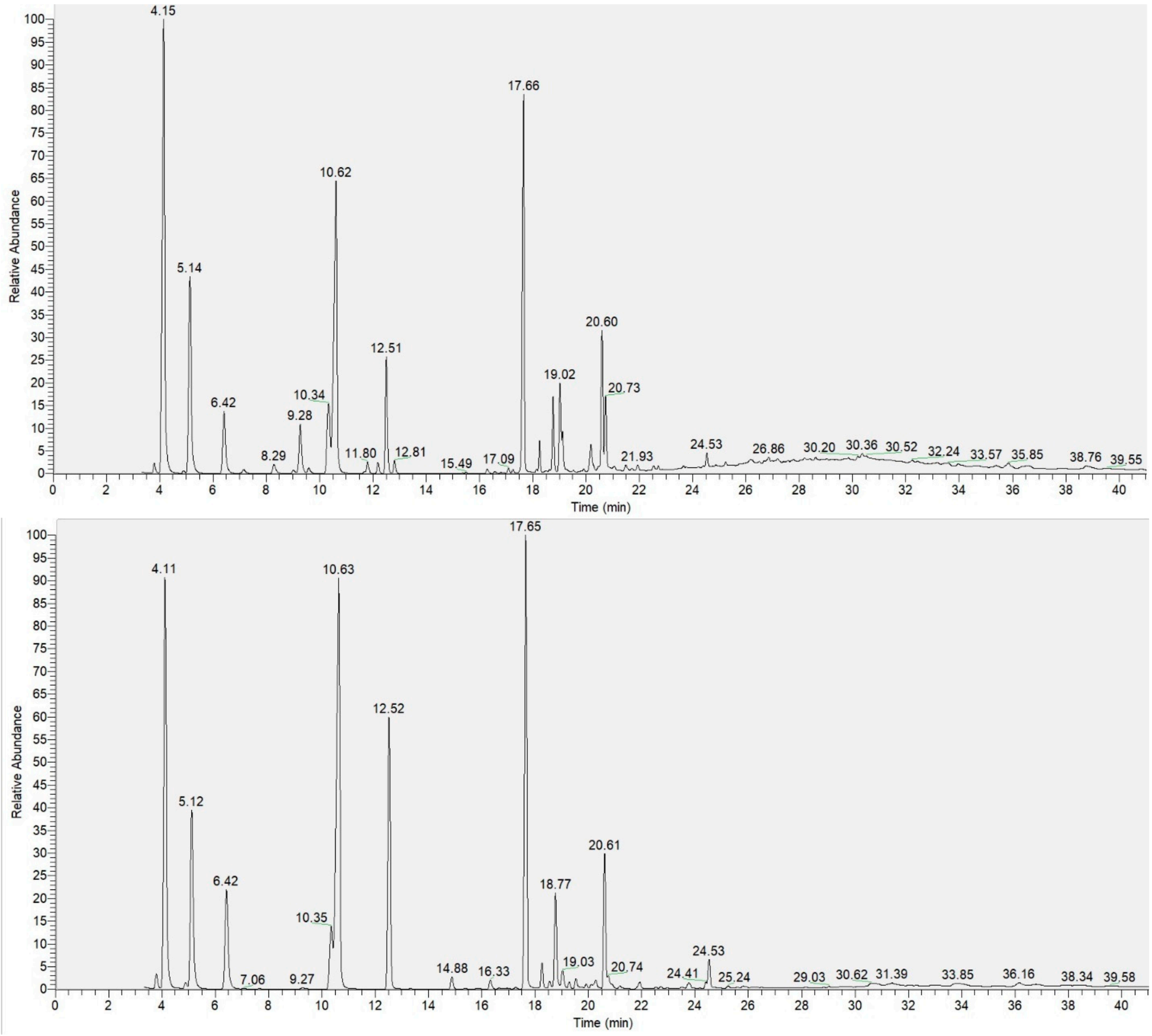

Figure S1. Chromatograms of Serbian (upper) and Russian (bottom) essential oils. TIC values for Serbian and Russian oils were 8.90 x 10^7 and 6.69 x 10^7, respectively
